# Effectiveness of Direct Antiviral Agents in People with HCV-Monoinfection Compared to HCV/HIV Coinfection in a Real Life Setting

**DOI:** 10.3390/v16111724

**Published:** 2024-10-31

**Authors:** Cristina Guadalupe Román López, Salma Triana González, Ana Luz Cano Díaz, Dulce Daniela Flores Lopez, José Antonio Mata Marín, Jesús Enrique Gaytán Martínez

**Affiliations:** 1 Internal Medicine Department, Hospital Regional No. 1 “Ignacio García Tellez”, Instituto Mexicano del Seguro Social, Mérida 97150, Yucatán, Mexico; cristinagromanl@gmail.com; 2 Infectious Diseases Department, Hospital de Infectologia “La Raza” National Medical Center, Instituto Mexicano del Seguro Social, Mexico City 02990, Mexico; stg.9204@gmail.com (S.T.G.); ana.knodiaz@gmail.com (A.L.C.D.); danis_club@hotmail.com (D.D.F.L.); jgaytanmtz@gmail.com (J.E.G.M.)

**Keywords:** viral hepatitis, therapy, Latin America

## Abstract

Direct-acting antivirals (DAA) are effective in patients with hepatitis C virus (HCV) infection, but there is little information about real-world effectiveness in people living with human immunodeficiency virus (PLH). The aim of this study was to determinate the effectiveness of DAA to achieve sustained virologic response at week 12 post-treatment (SVR12) in PLH with HCV coinfection and in people with HCV-monoinfection. We conducted a prospective cohort. The full analysis set (FAS) included all subjects enrolled in the study; the modified analysis set (MAS) excluded cases with missing data to evaluate SVR12. A total of 278 people were included, 130 (46.7%) with HCV/HIV-coinfection and 148 (53.2%) with HCV-monoinfection. In the HCV/HIV-coinfection group, 82 (63%) received GLE/PIB for 8 weeks, 45 (34.6%) received SOF/VEL for 12 weeks, and 3 (2.3%) were treated with SOF/VEL + RBV for 12 weeks. In the HCV-monoinfection group, 62 (41.8%) received GLE/PIB for 8 weeks, 28 (18.9%) received SOF/VEL for 12 weeks, and 58 (39.1%) participants were treated with SOF/VEL + RBV for 12 weeks. In the FAS analysis, SVR12 was 81.6% in the HCV/HIV-coinfection group and 86.4% in the HCV-monoinfection group (*p* = 0.128). In the MAS analysis, both groups achieved 100% of SVR12. In this cohort, the effectiveness of DAA to achieve SVR12 was similar between HCV/HIV-coinfection and HCV-monoinfection cases, regardless of advanced liver disease with no differences between treatment regimens.

## 1. Introduction

There are approximately 4–5 million people living with HIV (PLH) coinfected with hepatitis C virus (HCV) [1]. HCV prevalence in PLH and in men who have sex with men (MSM), is substantially higher than in the general population with 6.3% (5·14–7·29) [2].

Traditionally, the main risk factor for HCV was parenteral acquisition, with higher risk in drug users who share needles or sniffing equipment [3,4]. In MSM, HCV infection occurs in the setting of traumatic sexual practices, which commonly involves voluntary intake of certain psychoactive and non-psychoactive drugs in the context of sex parties and sexual intercourse with the intention to facilitate and/or enhance the sexual encounter, also known as chemsex [5].

The natural history of HCV infection is characterized by having spontaneous clearance in the absence of treatment within 6 months of infection in around 25% of cases [6]. The remaining 55–85% develop chronic infection, which can lead to progressive liver fibrosis and cirrhosis. The risk of cirrhosis in those with chronic hepatitis C infection ranges from 15% to 30% after about 20 years of infection with HCV [6].

On the other hand, HIV-related mortality has decreased since the advent of effective antiretroviral therapy (ART); liver disease has emerged as one of the most common non-AIDS-related causes of death among PLH [7]. Most cases of liver disease in this population are secondary to coinfection with hepatotropic viruses such as HCV and/or hepatitis B virus (HBV) [8]. HIV/HCV coinfection leads to accelerated hepatic fibrosis progression compared to patients with HCV-monoinfection [9]. Conversely, HCV may adversely affect HIV infection and reduce the effectiveness of ART [10].

Previously, a combination of pegylated interferon (PEG-IFN) and ribavirin (RBV) showed a lower rate of sustained virological response (SVR) and a higher rate of adverse events, and this was greater in those with HIV/HCV-coinfected patients compared to HCV-monoinfection [11]. Direct-acting antivirals (DAA) change the outlook for people with HCV infection, because of high efficacy to maintain SVR at 12 weeks post-treatment (SVR12). DAA inhibits specific HCV non-structural proteins (NS) that are important for viral replication [12]. Initially, DAA were indicated in combination with PegIFN-alpha and RBV, but these treatments were less effective for some genotypes [13].

In Mexico, we have two pangenomic regimens available; the first one is Sofosbuvir/Velpatasvir (SOF/VEL). SOF is a pyrimidine nucleotide analog inhibitor of NS5B in combination with VEL, an inhibitor of viral NS5A. A fixed-dose combination of VEL 100 mg and SOF 400 mg is to be taken orally once daily for 12 weeks or in combination with RBV in patients with decompensated cirrhosis [12]. The other pangenomic regimen is Glecaprevir (GLE), a NS3/4A inhibitor, coformulated with Pibrentasvir (PIB), an NS5A inhibitor. This regimen is also available as a fixed-dose tablet with GLE 100 mg and PIB 40 mg. Recommended doses are GLE 300 mg and PIB 120 mg daily for 8 weeks [14].

In phase 3 trials involving patients with HCV monoinfection, SOF/VEL once daily for 12 weeks provided high rates of SVR12 in HCV treatment with no prior experience to DAA and previously treated patients with any kind of genotype infection [15,16]. This regimen has also proved effective in real-world settings [17]. GLE/PIB has also shown >95% effectiveness for SVR12 in HCV monoinfection, irrespective of prior HCV treatment experience or cirrhosis diagnosis [18,19,20].

DAA regimens in PLH with HCV coinfection without cirrhosis, as SOF/VEL for 12 weeks or GLE/PIB for 8 weeks, are both safe and provide high rates of SVR12 in clinical trials [21,22]. In a phase 3, open-label ASTRAL-4 study, patients with decompensated cirrhosis caused by chronic HCV infection showed high rates of SVR12 in all treatment groups, including patients with genotype 3 infection. Previous studies that included data on this genotype reported lower rates of response than did patients with non-3 genotypes. In that pivotal trial, 85% effectiveness was achieved among patients with HCV genotype 3 who were treated with SOF/VEL plus RBV compared with the 71% response rate previously reported [23].

Some studies in real-world settings that evaluate the effectiveness of DAA reported a higher rate of SVR12 in HCV monoinfected compared with HCV/HIV. However, after adjustment for cirrhosis status, genotype, and type of treatment, living with HIV was not associated with a lower SVR12. In this study, liver cirrhosis was the only factor associated with a lower viral response [24].

In summary, data in real-life settings of DAA in HCV/HIV coinfection is limited. The aim of this study was to assess DAA effectiveness to achieve SVR12 in people with HCV/HIV coinfection compared with HCV-monoinfection.

## 2. Materials and Methods

A prospective cohort was conducted at the Hospital de Infectología “La Raza” National Medical Center, Mexico City, from January 2021 to December 2023. The HCV clinic is a third-level reference center for people with social security coverage.

The study was approved by Instituto Mexicano del Seguro Social, local health research committee 3502, and Research Ethics Committee 35028.

The criteria to start DAA was according to the hepatitis C program in Mexico, which does not differ from international guidelines. The treatment is provided to all treatment naïve and treatment experienced people with chronic HCV infection with or without cirrhosis, people with extrahepatic manifestations, after liver transplantation, people at risk of rapid progression of liver disease (HBV and HIV coinfection, diabetes, solid organ or stem cell transplant recipients), and people at risk of HCV transmission. Treatment is usually not recommended in people with limited life expectancy due to non–liver-related comorbidities. All the people data was uploaded to the HCV national platform.

### 2.1. Study Population

We included men and women, ≥18 years old, with HCV infection confirmed with HCV RNA viral load, living or not with HIV, with functional cure of HBV or under treatment with nucleotide analogue for HBV, with or without prior interferon (IFN) experience. DAA experience was permitted just if they had confirmed reinfection. People were excluded if they had DAA previous failure. All participants signed a written informed agreement.

### 2.2. Measurements

In the first medical visit, all patients were evaluated by a group of infectious diseases physicians to assess chronic hepatitis C confirmation, glomerular filtration rate, HIV and/or hepatitis B virus (HBV) coinfections, hepatocellular carcinoma, and staging of liver damage according to scores: Child–Pugh, AST to Platelet Ratio Index (APRI), and Fibrosis-4 Index (FIB-4), Model for End-Stage Liver Disease-Sodium (MELD-Na) score. The decision to treat was according to the infectious diseases physician who evaluated the patient and could decide to start SOF/VEL or GLE/PIB. Only in patients with Cirrhosis Child-Pugh B or C or with a history of liver decompensation, SOF/VEL plus RBV was started. In addition, when a PLH had in a regimen based on protease inhibitor (Darunavir/cobicistat), SOF/VEL was also initiated (to avoid drug interactions). Before the start of DAA, drug interactions between drugs used by patients and DAA were evaluated on the web page: https://www.hep-druginteractions.org/ (accessed on 1 January 2021).

Information was obtained during each medical visit; the first at the beginning of treatment, the second at the end of treatment (ETR), and a third to evaluate SVR12. If they had RBV in their regimen, medical visits were every month, or even more frequent, to detect adverse events related to RBV use, especially a decrease in hemoglobin. Collected data were age, gender at birth, HIV status, ART, history of IFN treatment or DAA, HCV RNA viral load, hemoglobin, platelet count, liver function, and coagulation tests. APRI, FIB-4 was calculated at the first medical visit, before starting DAA. Fibrosis through transient elastography (TE) was reported in kilopascals (kPa) by acoustic radiation force impulse (ARFI) technique (QelaXto) with multi-frequency convex transducer equipment by ultrasonography (US), performed by a single, experienced operator. The measurements were considered reliable with at least 10 successful acquisitions in kPa and an IQR less than 30% of median. TE results were classified as: <7.1 kPa (F0–F1), no liver fibrosis; 7.1–9.5 kPA (F2); ≥9.5–<12.5 kPa (F3), and ≥12.5 kPa (F4) or cirrhosis.

In PLH, CD4+ cell count and HIV-1 RNA viral load were also measured, prior to start DAA, at the end of treatment, and at SVR12. For the people with cirrhosis, we calculated the Child–Pugh score and Model for End-Stage Liver Disease-Sodium (MELD-Na) score. Adverse events to DAA and drug interactions were evaluated in each medical visit.

End of treatment response (ETR) was defined as undetectable HCV RNA viral load (<12 IU/mL) at the end of DAA treatment (8 weeks for GLE/PIB and 12 weeks for SOF/VEL with or without RBV); SVR12 was defined as undetectable HCV-RNA viral load 12 weeks after the end of DAA treatment. HCV RNA viral load was determined for each patient with quantitative PCR with lower limit of detection of <12 IU/mL with the Abbott Realtime HCV RNA viral load assay.

All patients with complete data were included at baseline. Three patients did not test blood samples at the time we requested, but they did it during the same month. Finally, for SVR12, two patients who did not test at the time were requested, but they also did it in the same month.

### 2.3. Statistical Analysis

Sample size was obtained with non-probability sampling. Kolmogorov–Smirnov test was used to determine data distribution. Descriptive results were summarized using median and interquartile ranges (IQR) for a non-normal distribution. Effectiveness was assessed by the percentage of patients achieving SVR12 with two analyses as previously stated in real-world studies. The full analysis set (FAS) included data from all patients enrolled in the study; the modified analysis set (MAS) included only the patients with missing values at the time point to evaluate SVR12 [20]. Comparison of demographic characteristics and biochemical baseline data (prior to treatment) and at two measures after DAA treatment (ETR and SVR12) between HCV-monoinfection and HCV/HIV-coinfection groups was done, using the Chi-square test or Fisher’s exact test for qualitative data and the Mann–Whitney U test for quantitative data. A *p* value < 0.05 was considered statistically significant for differences among groups. All analyses were made with SPSS software (version 26; BM^®^ SPSS^®^ Corp., Armonk, NJ, USA).

## 3. Results

A total of 278 people with HCV infection were included: 87 (58.7%) women and 61 (41.2%) men in the HCV group, and 11 (8.4%) women and 119 (91.5%) men in the HCV/HIV group; 130 (46.7%) participants had HCV/HIV coinfection; the median age was 35 years (IQR 29–41) for the HCV/HIV coinfected group and 58 years (IQR 47–69) for the HCV monoinfected group, *p* < 0.001. Cirrhosis was found in 84 (56.7%) cases in the HCV group and 12 (9.2%) cases in HCV/HIV group, *p* < 0.001. Median of kPa by elastography was 14.7 (IQR 3.2–26.2) kPa in the HCV group and 6 (IQR 4.95–7.05) kPa in HIV/HCV group, *p* < 0.001.

All PLH had ART, and the most frequent regimen contained was a second-generation integrase inhibitor (INSTI). All of them with HIV-1 RNA viral load undetectable (<40 copies/mL) and median CD4+ cells count of 572 (IQR 413–731) cells/mm3. Baseline RNA HCV viral load was 793,125 (IQR 198,734–1,190,593) IU/mL in HCV group and 1,687,026 (IQR 843,516–2,530,542) IU/mL in HCV/HIV group, *p* < 0.001, (Table 1).

The baseline biochemical characteristics at the beginning of the treatment and at the moment of the SVR12 show an improvement in both groups. The HCV RNA viral load at the end of the treatment was not detectable in both groups (Table 2).

Of the 278 participants, 44 patients lost follow-up because they changed institutions, mainly because they lost their job and consequently, their social security coverage; two because they moved to another state, and two of them migrated from our country. SVR12 was achieved in 106 (81.6%) participants in the HCV/HIV group; of them, 82 (63%), 45 (34.6%), and 3 (2.3%) participants were treated with GLE/PIB during 8 weeks, SOF/VEL during 12 weeks, and SOF/VEL + RBV during 12 weeks, respectively. In the HCV group, SVR12 was observed in 128 (86.4%) participants, 62 (41.8%) had GLE/PIB during 8 weeks, 28 (18.9%) had SOF/VEL during 12 weeks, and 58 (39.1%) were treated with SOF/VEL + RBV during 12 weeks. Only four (2.6%) participants in the HCV group experienced severe adverse events, and only one (0.35%) woman on GLE/PIB therapy prematurely discontinued treatment for skin symptoms but achieved SVR12 (Table 3).

In the FAS analysis, SVR was achieved in 106 (81.6%) participants in the HCV/HIV coinfection group compared with 127 (86.4%) participants in the HCV-monoinfection group (*p* = 0.128). In the MAS analysis, we included 234 people, 106 (45.2%) with HIV/HCV coinfection and 148 (54.7%) with HCV, and all the people in both groups achieved 100% of SVR12.

## 4. Discussion

In this prospective cohort, we found that the effectiveness of DAA to achieve SVR12 in people with HCV/HIV-coinfection and in subjects with HCV-monoinfection was similar. For effectiveness, we performed two types of analyses. In FAS analysis there was not a significant difference between groups, and in the MAS analysis all subjects achieved SVR12. None of the demographic characteristics, baseline biochemical alterations, or regimen of DAA affected virologic response in our population.

Regarding clinical and biochemical characteristics between groups, we found some differences. There were more women, older people, and a higher proportion of cirrhosis defined by elastography in the HCV-monoinfection group. This was also reflected in the baseline liver function test with a lower level of albumin, platelets count, and a higher level of total bilirubin. On the other hand, aminotransferases were higher in HCV/HIV-coinfected patients, particularly ALT. After treatment with DAA, the aminotransferases level decreased in both groups and the level of platelets increased. These demographic and clinical differences might be a result of late diagnosis in HCV-monoinfection patients. Most of the women were tested when they already had cirrhosis. Young men are tested more frequently as a result of programs related to control of sexually transmitted diseases [5]. This disparity in missing opportunities of diagnosis is likely related to a reduced scope of screening programs in our country.

In an epidemiological study that took place in another region of Mexico, the advanced liver fibrosis prevalence was highest in patients with HIV/HCV co-infection (47.7%), especially in those with HCV subtype 1a. CD4+ counts, albumin, direct bilirubin, and indirect bilirubin were associated with liver fibrosis, probably due to sociodemographic factors such as social stratum, level of education, and late diagnosis of HCV [25].

In this population, HCV-monoinfected patients reported a higher proportion of adverse events to DAA, probably related to older age, more advanced liver disease, increased use of drugs due to comorbidities, and use of RBV in association with DAA due to decompensated cirrhosis. RBV can cause severe hemolytic anemia in up to 10% of patients, requiring monitoring of hemoglobin while on treatment and frequent dose adjustments of the drug. Other reported side effects of RBV included itching, fatigue, and upper respiratory symptoms [26].

Regarding effectiveness, all participants in this study had an undetectable HCV RNA viral load at the end of the treatment. In addition, we also observed a decrease in the level of transaminases. Furthermore, SVR was reached in all patients in whom HCV RNA viral load determination was available 12 weeks after completion of treatment.

Compared with other studies, we did not have a treatment failure in the MAS analysis. In a cohort that took place in Egypt, they reported treatment failures, although the rate of SVR12 did not differ significantly between both groups (95.9% versus 97.3% in coinfected and monoinfected groups, respectively; *p* > 0.05). The factors associated with worse response to therapy in that study were male sex and having genotype 3. Although factors associated with worse response to therapy occurred more often in the HIV coinfection group, real-life results of DAA did not differ significantly between both populations [27]. Compared with this cohort, we found fewer patients with previous experience with HCV treatments. Also, we did not perform pre-treatment genotypes; although, according to previous published data, genotype 1 is the most frequent in Mexico and usually responds very well with both regimens of DAA [5,25,26]. Another point to consider that could explain the absence of virologic failure in MAS analysis of our population is the type of DAA. In older studies, used regimens of DAA were less effective and more frequently affected by baseline resistance-associated substitutions (RAS) [28].

In this study, people were only treated with GLE/PIB and SOF/VEL (ribavirin was added to the SOF/VEL regimen in case of history of decompensated cirrhosis or cirrhosis Child Pugh B or C), in contrast with other cohorts that include several regimens, some of them with less effectiveness [29]. In the FAS analysis, we did not have the HCV viral load to confirm the SVR12 in some cases who were lost to follow-up, mainly because patients lost social security coverage, or they moved and got medical attention in another HCV clinic.

In this cohort, differences were not found in SVR12 rate between the two regimens. The decision to start DAA (SOF/VEL or GLE/PIB) was done by the clinical team, taking in consideration if the patients had liver failure or protease inhibitor as part of the ART. In that case, GLE/PIB was not recommended because of the potential for drug-drug interactions [30].

Data in this study is also in concordance with a retrospective cohort in Spain, where a group of people with HCV/HIV coinfection and another with HCV-monoinfection were treated with GLE/PIB. Patients achieved SVR12 rates greater than 95%. The response was similar in HIV/HCV-coinfected people and HCV-monoinfected people. Regarding cirrhosis and non-cirrhotic people, both achieved similar rates of SVR12 [31].

Similarly, another study that assessed the efficacy and safety of SOF/VEL in HCV and HIV patients with coinfection on dolutegravir-based ART found that 42 (97.7%) of the people attained SVR12; this could not be assessed in two patients. SVR-12 rate was 97.7% and 93.3%, per protocol and intention to treat analysis, respectively, with no grade III/IV adverse events reported that led to discontinuation [32]. In our cohort, only one woman in the HCV monoinfected group experienced adverse events that led to discontinuation of GLE/PIB after 4 weeks of treatment because of rash and skin problems. But at the same, time the patient started several alternative drugs not approved in Mexico, so adverse events cannot be fully explained by DAA. Still, SVR12 was achieved with 4 weeks of treatment.

As we previously stated, we found high rates of SVR (100%) where we did not have loss of follow-up. Some studies have demonstrated that active drug users could have decreased SVR, both in HCV/HIV coinfection and HCV monoinfection, independently of baseline characteristics, mainly due to voluntary dropout [31,33]. Despite the fact that in the Central Mexico region there is a high prevalence of drug use in PLH that could lead to voluntary dropout of DAA, mainly drugs used in chemsex, such as ethyl chloride and poppers, intravenous drugs and other kinds of illicit drugs are not common [5].

The study has several limitations. HCV genotype was not available prior to treatment initiation to let us better understand the viral factors that influence treatment response. Also, many patients lost follow-up, and this limits analysis of treatment effectiveness. The patients attending the hospital are people with social security. If they lose their coverage, they cannot be attended in this institution anymore, and that explains the high rate of patients that were lost due lack of follow-up that was found in this cohort.

The study has some strengths. First, we were able to evaluate the effectiveness of two different regimens of DAA to achieve SVR12 in PLH and people without HIV, under real-world conditions. In addition, elastography measurements were performed in most of the patients. This diagnostic approach for fibrosis is not widely available in Mexico, and this allowed us to make a more objective fibrosis categorization in our population. Finally, we performed a viral load determination immediately after the subject finished DAA that helped us differentiate reinfection from treatment failure in high-risk populations for hepatitis C reinfection, as PLH with sexual risk factors, intravenous drug, and chemsex users.

## 5. Conclusions

The effectiveness of DAA is high in people with HCV infection and people with HIV/HCV coinfection. Both groups of patients achieved SVR12 despite the epidemiological and clinical differences between groups. Pangenomic regimens showed a high level of safety for both groups, and only the use of RBV was associated with more severe adverse events.

There is a need for improving screening programs for hepatitis C infection, mostly in vulnerable populations to avoid the consequences of chronic infection and liver failure.

## Figures and Tables

**Table 1:** Baseline characteristic of the study population (N = 278).

	HIV/HCV (n = 130) Median (IQR) or (%)	HCV (n = 148) Median (IQR) or (%)	*p*-Value
Age (years)	35 (IQR 29–41)	58 (IQR 47–69)	<0.001
Sex			
Men	119 (91.5%)	61 (41.2%)	
Women	11 (8.4%)	87 (58.7%)	<0.001
Cirrhosis	12 (9.2%)	84 (56.7%)	<0.001
Child Pugh			0.035
A	11 (8.3%)	44 (29.7%)	
B	1 (0.75%)	32 (21.6%)	
C	0	8 (5.4%)	
MELD-Na	8 (IQR 6.5–9.5)	10 (IQR 7.0–13)	0.219
APRI, median	0.7 (IQR 0.3–1.0)	1.1 (IQR 0.1–2.3)	0.011
FIB-4, median	0.96 (IQR 0.56–1.36)	3.75 (IQR 1.18–6.32)	<0.001
Elastography (kPa)	6 (IQR 4.95–7.05)	14.7 (IQR 3.2–26.2)	<0.001
F0–F1	94 (72.3%)	35 (23.6%)	
F2	8 (6.1%)	11 (7.4%)	
F3	7 (5.3%)	6 (4%)	
F4	10 (7.6%)	63 (42.5%)	
Antiretroviral treatment	132 (100%)	N/A	N/A
Type of ART		N/A	N/A
BIC/TAF/FTC	105 (80.7%)		
DTG/ABC/3TC	13 (10.0%)		
DTG + DRV/c + TDF/FTC	6 (4.6%)		
DRV/c + TDF/FTC	3 (2.3%)		
DRV/c + DTG	2 (1.5%)		
DTG + 3TC	1 (0.7%)		
Undetectable HIV-1 RNA viral load (<40 copies/mm^3^)	130 (100%)	N/A	N/A
CD4+ cells count (cells/mm^3^)	572 (IQR 413–731)	N/A	N/A
HCV RNA viral load (IU/mL)	1687026 (IQR 843,516–2,530,542)	793125 (IQR 198,734–1,190,593)	<0.001

HCV: hepatitis C virus. HIV: human immunodeficiency virus. kPa: kilopascals. APRI: aspartate aminotransferase to platelet ratio index. FIB-4: fibrosis-4 index for liver fibrosis. BIC: Bictegravir. TAF: Tenofovir alafenamide. FTC: Emtricitabina. DTG: Dolutegravir. ABC: Abacavir. 3TC: Lamivudina. DRV/c: Darunavir/cobicistat. TDF: Tenofovir fumarate. RNA: ribonucleic acid. MELD-Na: Model for End-Stage Liver Disease-Sodium. N/A: “not applicable”.

**Table 2:** Biochemical characteristics, N = 278.

	HIV/HCV (n = 130) Median (IQR) or (%)	HCV (n = 148) Median (IQR) or (%)	*p*-Value
Determination Before DAA treatment			
AST (U/L)	61 (IQR 26.5–96.5)	57 (IQR 26–88)	0.004
ALT (U/L)	87 (IQR 28–146)	51 (IQR 23–79)	<0.001
TB (mg/dL)	0.7 (IQR 0.4–1)	1 (IQR 0.45–1.4)	0.002
DB (mg/dL)	0.3 (IQR 0.15–0.45)	0.4 (IQR 0.15–0.55)	0.016
IB (mg/dL)	0.4 (IQR 0.25–0.55)	0.5 (0.3–0.7)	0.001
Albumin (g/dL)	4.3 (IQR 4.0–4.3)	3.9 (IQR 3.3–4.5)	<0.001
Platelets (10 + 3/microL)	248 (IQR 199–296)	141 (IQR 68–214)	<0.001
INR	0.92 (IQR 0.87–0.97)	0.98 (IQR 0.9–1.06)	0.494
TP (seg)	11.2 (IQR 10.55–11.85)	12.1 (IQR 11.1–13.1)	<0.001
Determination at the end of the treatment			
	HIV/HCV (n = 122) Median (IQR) or (%)	HCV (n = 138) Median (IQR) or (%)	*p*-value
AST (U/L)	24 (IQR 14–34)	25 (IQR 21–28)	0.095
ALT (U/L)	23 (IQR 12–34)	20 (IQR 14–26)	0.057
TB (mg/dL)	0.8 (IQR 0.6–1)	0.8 (IQR 0.3–1.3)	0.304
DB (mg/dL)	0.3 (IQR 0.2–0.4)	0.38 (IQR 0.18–0.58)	0.475
IB (mg/dL)	0.5 (IQR 0.35–0.65)	0.5 (0.3–0.7)	0.057
Albumin (g/dL)	4.4 (IQR 4.25–4.45)	4.4 (IQR 4.1–4.7)	0.139
Platelets (10 + 3/microL)	218 (IQR 177–259)	180 (IQR 127–233)	<0.001
INR	0.93 (IQR 0.88–0.98)	0.95 (IQR 0.85–1.06)	0.034
PT (seg)	11.0 (IQR 10.0–2.0)	11.5 (IQR 10.0–13.0)	0.016
Undetectable HCV RNA viral load (IU/mL)	122 (100%)	138 (100%)	0.133
Determination during SVR 12 weeks			
	HIV/HCV (n = 106) Median (IQR) or (%)	HCV (n = 128) Median (IQR) or (%)	*p*-value
AST (U/L)	24 (IQR 17–31)	29 (IQR 19.5–38.5)	0.780
ALT (U/L)	19 (IQR 13–25)	23 (IQR 16.5–30.5)	0.560
TB (mg/dL)	0.8 (IQR 0.55–1.05)	0.7 (IQR 0.2–1.2)	0.050
DB (mg/dL)	0.3 (IQR 0.2–0.4)	0.39 (IQR 0.1–0.5)	<0.001
IB (mg/dL)	0.46 (IQR 0.31–0.61)	0.5 (IQR 0.2–0.8)	0.361
Albumin (g/dL)	4.5 (IQR 4.25–4.75)	4 (IQR 3.55–4.45)	<0.001
Platelets (10 + 3/microL)	243 (IQR 199–287)	122 (IQR 61.5–180.5)	<0.001
INR	0.91(IQR 0.86–0.96)	1 (IQR 0.94–1.06)	<0.001
PT (seg)	11 (IQR 10.35–11.65)	12.1 (IQR 11.15–13.05)	0.350
Undetectable HCV RNA viral load (IU/mL)	106 (100%)	128 (100%)	0.324

AST: aspartate transaminase. ALT: alanine transaminase. TB: total bilirrubin. DB: direct bilirubin. IB: indicrect bilirubin. INR: International normalized ratio. PT: prothrombin time. HIV: human immunodeficiency virus. HCV: hepatitis C virus. kPa: kilopascals. APRI: aspartate aminotransferase to platelet ratio index. FIB-4: fibrosis-4 index for liver fibrosis. RNA: ribonucleic acid.

**Table 3:** Effectiveness and safety of DAA at SVR12 (n = 278).

	HIV/HCV (n = 130) Median (IQR) or (%)	HCV (n = 148) Median (IQR) or (%)	*p*-Value
SRV 12 weeks (FAS)	106 (81.6%)	128 (86.4%)	0.128
GLE/PIB 8 weeks	82 (63.0%)	62 (41.8%)	
SOF/VEL 12 weeks	45 (34.6%)	28 (18.9%)	
SOF/VEL + RBV 12 weeks	3 (2.3%)	62(41.8%)	
SVR 12 weeks (MAS) (n = 234)	106 (100%)	128 (100%)	0.142
GLE/PIB 8 weeks	69 (53.07%)	52 (35.1%)	
SOF/VEL 12 weeks	35 (26.9%)	26 (17.4%)	
SOF/VEL + RBV 12 weeks	2 (1.5%)	50 (33.7%)	
Undetectable HCV RNA viral load (IU/mL)	106 (100%)	128 (100%)	0.325
Type of treatment			
No prior treatment	116 (89.2%)	139 (93.9%)	0.157
Experienced to treatment	14 (10.7%)	9 (6.0%)	
DAA	12 (9.2%)	1 (0.6%)	<0.001
IFN	2 (1.5%)	8 (5.4%)	0.084
Adverse events	81 (62.3%)	129 (87.1%)	<0.001
Severe adverse events that did not lead to discontinuation	0 (0%)	4 (2.7%)	0.065
Severe adverse events that led to discontinuation	0 (0%)	1 (0.67%)	0.247

SVR12: sustained virologic response at week 12. HIV: human immunodeficiency virus. HCV: hepatitis C virus. GLE: Glecaprevir. PIB: pibrentasvir. SOF: Sofosbuvir. VEL: Velpatasvir. RBV: ribavirin. DAAs: direct-acting antivirals. IFN: interferon. RNA: ribonucleic acid.

## Data Availability

The original contributions presented in the study are included in the article; further inquiries can be directed to the corresponding author.
